# Systemic steroid application for treatment of edematous anastomotic stenosis following delta-shaped anastomosis in laparoscopic distal gastrectomy: a case report

**DOI:** 10.1186/s12893-020-00827-3

**Published:** 2020-07-22

**Authors:** Jun Arima, Kohei Taniguchi, Toshihiro Kobayashi, Ichiro Tsunematsu, Shuji Kagota, Junna Sakane, Yusuke Suzuki, Kazuhisa Uchiyama, Masako Hiramatsu

**Affiliations:** 1 Takatsuki Red Cross Hospital, 1-1-1 Abuno, Takatsuki, Osaka, 569-1096 Japan; 2 Department of General and Gastroenterological Surgery, Osaka Medical College, 2-7 Daigaku-machi, Takatsuki, Osaka, 569-8686 Japan; 3 Translational Research Program, Osaka Medical College, 2-7 Daigaku-machi, Takatsuki, Osaka, 569-8686 Japan

**Keywords:** Steroid, Delta-shaped anastomosis, Anastomotic stenosis, Gastrectomy, Gastric cancer

## Abstract

**Background:**

Delta-shaped anastomosis is a common method of intracorporeal gastroduodenostomy in totally laparoscopic distal gastrectomy. One common postoperative complication of this procedure is anastomotic stenosis, and endoscopic balloon dilatation is a major remedy for such complications. Other treatment strategies are necessary to manage unsuccessful endoscopic balloon dilatation.

**Case presentation:**

We present a case where systemic steroid treatment was applied in sustained anastomotic stenosis after endoscopic balloon dilatation. We performed delta-shaped anastomosis in laparoscopic distal gastrectomy to treat early-stage gastric cancer in a patient. The patient experienced abdominal pain post-surgery; subsequent investigation revealed edematous anastomotic stenosis. The stenosis sustained even after endoscopic balloon dilatation and local steroid injection. Consequently, we applied systemic steroid treatment.

**Conclusion:**

Systemic steroid treatment improved the stenosis and no recurrence was observed. These results suggest that systemic steroid application could be useful to treat anastomotic stenosis.

## Background

Surgical resection is the mainstay treatment for gastric cancer. Billroth-I is considered a simple reconstruction procedure, as it requires only one anastomosis in distal gastrectomy. Delta-shaped anastomosis is a common method of intracorporeal gastroduodenostomy in totally laparoscopic distal gastrectomy (Additional file 1) [1]. Anastomotic stenosis is a well-known postoperative complication associated with delta-shaped anastomosis [2], and endoscopic balloon dilatation (EBD) is a major remedy for such a complication [3]. In case of unsuccessful EBD, additional surgical management is required, resulting in a great burden on patients undergoing the procedure. To date, a solid alternative therapy for such surgical procedures has not been established, necessitating the development of non-surgical treatments for anastomotic stenosis. Here, we present a case where systemic steroid application was effective in sustained anastomotic stenosis after EBD.

## Case presentation

**Fig. 1:**
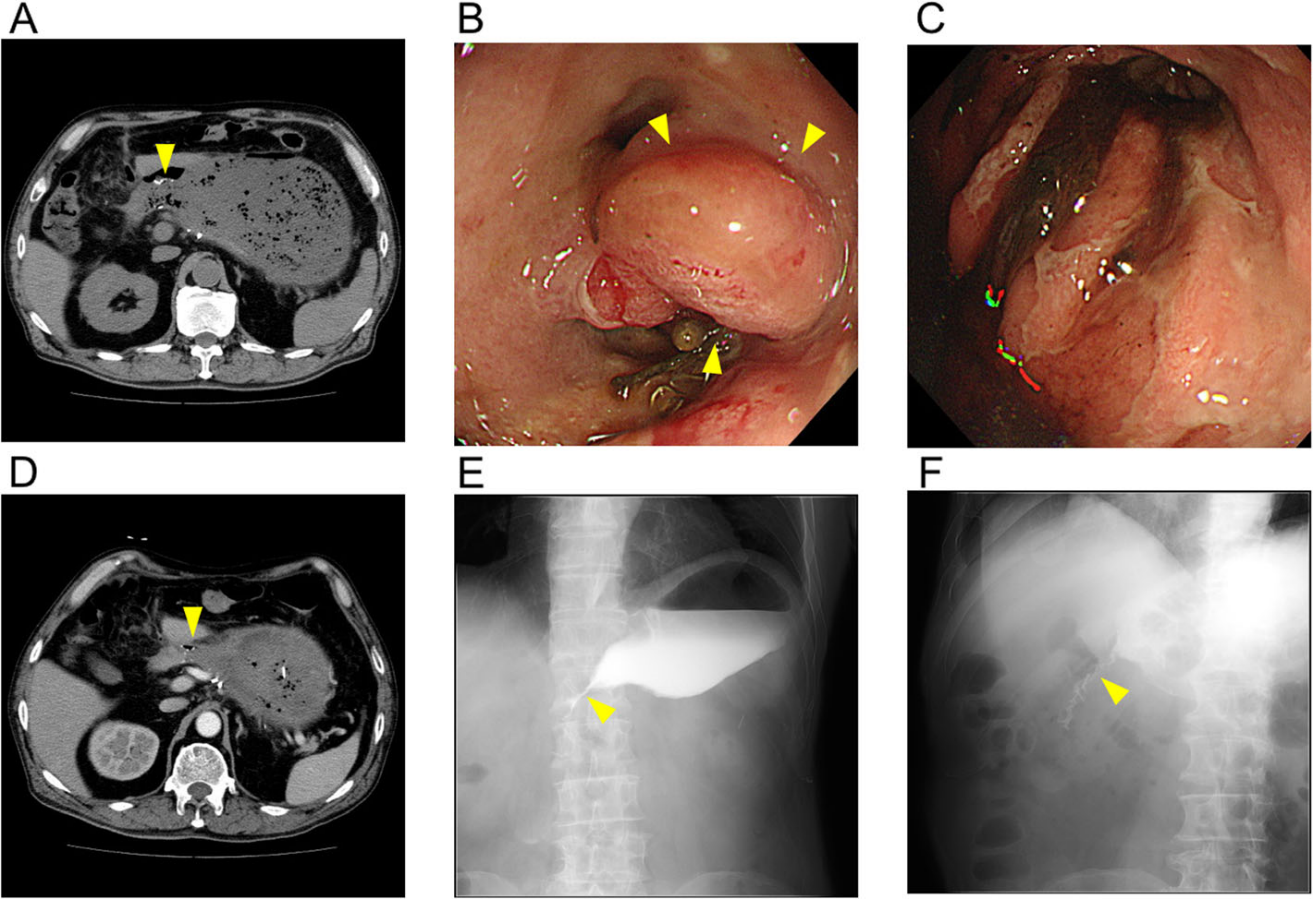
Representative images before systematic steroid treatment. **a** Computed tomography (CT) scans on postoperative day 9. The yellow arrowhead indicates the anastomotic part. **b** Endoscopy showing extreme swelling, like a polyp, in the duodenum on postoperative day 19. Yellow arrowheads indicate swelling. **c** Endoscopy showing the remaining portion of the stomach was edematous on postoperative day 19. **d** CT scan shows the wall of the anastomotic part was edematous, and swelling persisted on postoperative day 20. The yellow arrowhead indicates the anastomotic part. **e** Fluoroscopy revealing little contrast medium could flow into the duodenum on postoperative day 26. The yellow arrowhead indicates the contrast medium. **f** The quantity of contrast medium that could flow into the duodenum did not change after local injection of steroid on postoperative day 56. The yellow arrowhead indicates the contrast medium

**Fig. 2:**
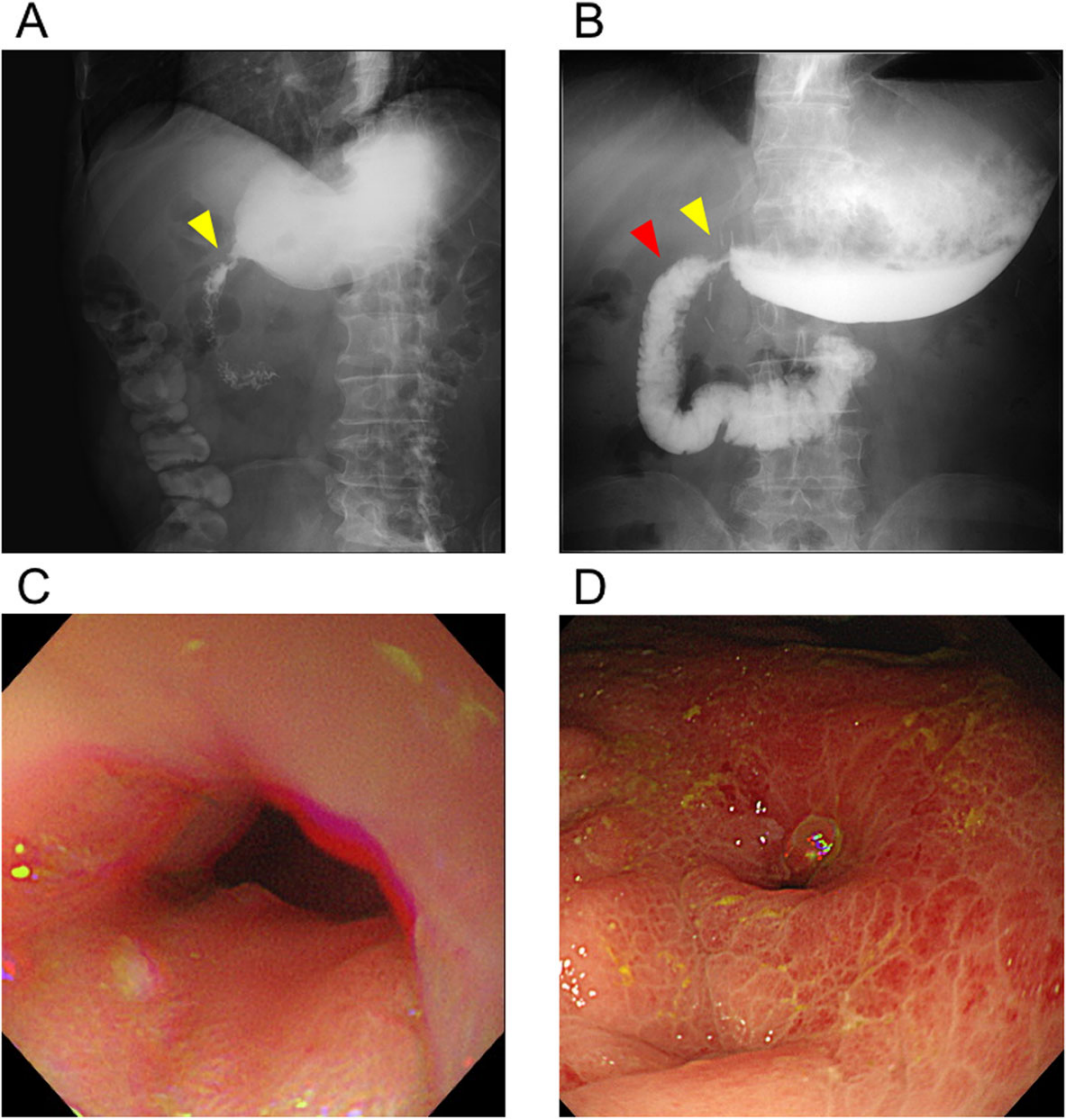
Representative images after systemic steroid treatment. **a** The quantity of contrast medium that could flow into the duodenum slightly increased on post-steroid treatment day 1 (postoperative day 57). The yellow arrowhead indicates the contrast medium. **b** Although partial anastomotic stenosis could be observed, the quantity of contrast medium that flowed into the duodenum definitely increased on post-steroid treatment day 37 (Postoperative day 93). Yellow and red arrowheads indicate the contrast medium. **c** The swelling of the duodenum disappeared on post-steroid treatment day 121 (Postoperative day 177). **d** The edema in the remaining part of the stomach had reduced on post-steroid treatment day 121 (Postoperative day 177)

**Fig. 3:**
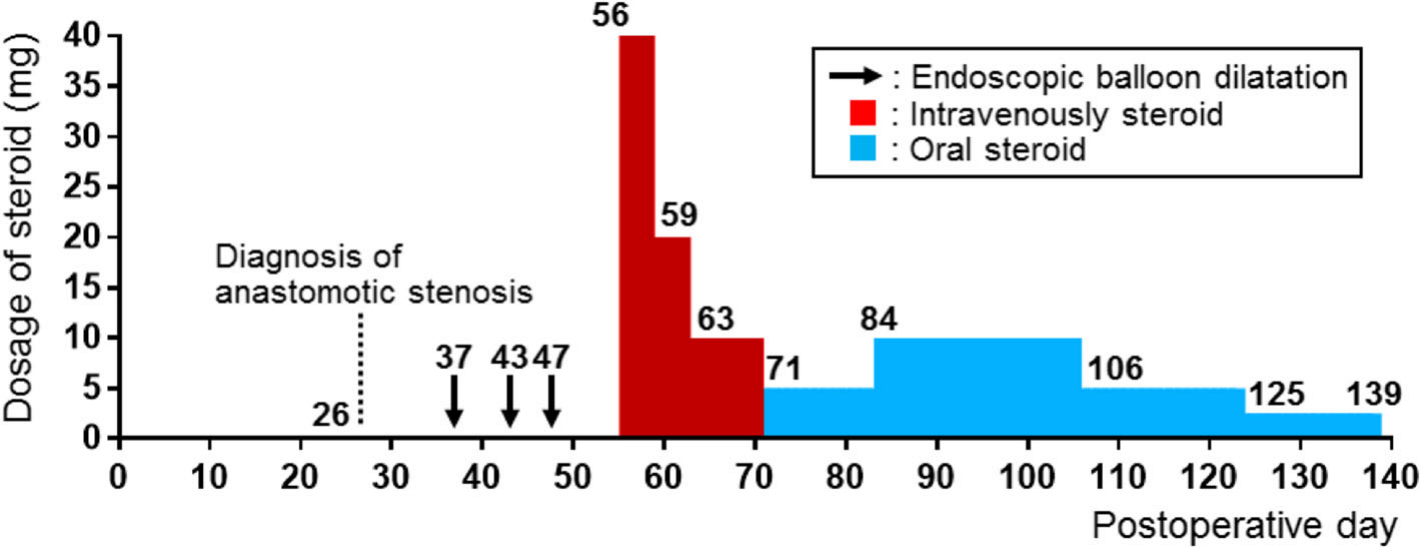
Time-series analysis of the treatment strategy. Anastomotic stenosis was diagnosed by fluoroscopy on postoperative day 26. EBD was performed on postoperative days 36, 43, and 47 (black arrows). Local steroid injection was performed alongside EBD on postoperative day 47. Systemic steroid treatment started on postoperative day 56. The oral administration of steroids started on day 15 (Postoperative day 71). Systemic steroid treatment finished on post-steroid treatment day 83 (postoperative day 139). The red and blue bars indicate periods of intravenous and oral steroid treatments, respectively

In March 2018, a 72-year-old man was referred to our department for surgical treatment of early-stage gastric cancer. Laparoscopic distal gastrectomy was performed by the Billroth-I producer using delta-shaped anastomosis. The posterior walls of both the stomach and the duodenum were joined using a 45-mm linear stapler in delta-shaped anastomosis. The enterotomy was closed with a 60-mm linear stapler. The patient consumed food and water between postoperative days 4 and 9. On postoperative day 9, the patient experienced left abdominal pain. Computed tomography (CT) scans revealed that the luminal continuity of the anastomotic part was wide enough for the passage of food (Fig. 1a). However, on postoperative day 19, an endoscopic investigation revealed anastomotic stenosis with severe edema (Fig. 1b, c). Contrast-enhanced CT scans also revealed that the anastomotic part was narrower compared to that observed in previous CT scans (Fig. 1d). Fluoroscopy clearly showed the presence of anastomotic stenosis (Fig. 1e). EBD was performed with 10 mm, 12 mm, and 15 mm-balloons approximately every week to treat the anastomotic stenosis. The stenosis sustained even after the injection of steroids into the anastomosis section during the third EBD (Fig. 1f). Consequently, we started systemic steroid treatment on postoperative day 56. For the first 4 days, the steroid (prednisolone) was injected intravenously at a dose of 40 mg/day. Stenosis started to improve soon after the onset of steroid treatment (Fig. 2a). Hence, for the next 3 and 8 days, the systemic steroid treatment was continued at a lower dosage of 20 mg/day and 10 mg/day, respectively. Thereafter, we started oral administration of steroids (5 mg/day). Although oral steroid administration was temporally increased (10 mg/day for 3 weeks) due to reappearance of nausea, fluoroscopy on postoperative day 93 showed improvement of anastomotic stenosis (Fig. 2b). Steroid treatment was discontinued for 3 months after completion of systemic steroid treatment. Endoscopic analysis approximately 1 month after completion of systemic steroid treatment showed improvement in anastomotic stenosis. (Fig. 2c, d). A summary of the EBD and steroid treatments is shown in Fig. 3. No dietary restrictions were required for 6 months following the completion of systemic steroid treatment. Moreover, no adverse events were found to be associated with steroid treatment.

## Discussion and conclusion

To the best of our knowledge, this case report is the first in PubMed to show that systemic steroid treatment could improve edematous anastomotic stenosis following gastrectomy with delta-shaped anastomosis. This case report also showed that multi-dose administration of systemic steroids could improve stenosis after EBD.

A previous study showed that local steroid injection in combination with EBD was effective in treating stenosis after esophagectomy [4], and in our case, we adapted the same methodology to treat stenosis after gastrectomy. However, in our case, local steroid injection was not effective in treating edematous anastomotic stenosis. This could be due to the fact that the edema was severe and, consequently, it was impossible to inject sufficient levels of steroids. A previous study reported that systemic steroid application was more effective than local steroid injection in treating stenosis after large endoscopic submucosal dissection of the stomach [5]. Systemic steroid administration immediately improved edematous anastomotic stenosis in 6 patients reported from Japan [6–8]. We have summarized the details of these cases, including ours in Table 1. Based on this evidence, we hypothesized that systemic steroid administration could be effective in treating edematous anastomotic stenosis, and consequently, we used steroid therapy as a secondary option along with EBD. The stenosis started to improve soon after the onset of steroid treatment. Such an observation is in line with a previously published report that showed steroids can not only inhibit collagen synthesis, but also enhance collagen degradation, thereby inhibiting stenosis formation [9]. Such a mechanism could induce long-term benefits by expanding the stenotic part after the completion of systemic steroid treatment.

**Table 1:** Details of the cases that underwent successful steroid treatment for anastomotic stenosis

Case number	Author (Year)	Sex	Age	Anastomotic location	Condition^a^	Anastomosis method^b^	EBD	Administration method	Steroid dose
Case 1	Satomi et al. (2018) [6]	Male	72	Transverse colon	Edema	Stapler	–	Local injection, Systemic steroid	Multi-dose
		Male	72	Sigmoid colon	Edema	Stapler	–	Systemic steroid	Multi-dose
		Female	24	Sigmoid colon	Edema	Stapler	–	Systemic steroid	Multi-dose
		Female	36	Transverse colon	Edema	Manual	–	Systemic steroid	Multi-dose
Case 2	Tsuneda et al. (2019) [7]	Male	68	Transverse colon	Edema	Manual	–	Local injection, Systemic steroid	Multi-dose
Case 3	Fujihata et al. (2017) [8]	Female	79	Stomach, duodenum	Edema	Stapler	–	Systemic steroid	Multi-dose
Case 4	Our case (2019)	Male	72	Stomach, duodenum	Edema	Stapler	+	Local injection, Systemic steroid	Multi-dose

Cases 1–3 were reported in Japanese

*EBD* endoscopic balloon dilatation

^a^: endoscopic appearance of anastomotic part, ^b^: the method of anastomosis on enterotomy

The edematous stenosis could have resulted from several factors such as tension created during anastomosis, local tissue ischemia, subclinical leakage, injury from acid exposure, and submucosal hematoma created during suturing [10]. In the present case, endoscopic and CT investigations showed that the main cause of anastomotic stenosis was not structural (such as the small lumen or twists in the anastomotic part) but primarily the formation of edema. While the mechanism of the observed edematous change remained unclear, the improvement of the edema was important in treating stenosis in this case. Based on these conditions, systemic steroid treatment could be safer with a more immediate effect than surgical re-approach. In this case, the extremely edematous remnant stomach increased the risk of re-anastomotic stenosis or leakage in gastrojejunostomy. Moreover, in the rhinoplasty, a meta-analysis concluded that multi-dose steroid administration was more effective in reducing edema than single-dose steroid application [11, 12]. Previous Japanese case reports have also mentioned the use of multi-dose steroid therapy in all cases (Table 1). Although even a single-dose of steroids may reduce edema compared with controls (no steroid administration), we employed multi-dose steroid therapy in the present case based on previous evidence.

However, several issues need to be considered. First, it should be noted that multi-dose steroid administration could lead to adverse events [13]. As such, the dosage of steroids should be as low as possible to prevent such events. Fortunately, in our case, we did not observe any side effects of steroid therapy. Second, it is necessary to have experience regarding single-dose steroid administration for anastomotic stenosis. Based on these issues, further studies are required with a large number of cases to determine the adequate dosages and time-periods for systemic steroid application to treat anastomotic stenosis. Third, although the prediction of anastomotic strictures is difficult, we may need to consider the earlier use of steroids to prevent worsening of anastomotic stenosis before EBD.

In conclusion, anastomotic stenosis is one of the postoperative complications associated with delta-shaped anastomosis in gastrectomy. Systemic steroid application could be useful in treating edematous anastomotic stenosis.

## Supplementary information

**Additional file 1.** The illustration of delta-shaped anastomosis in totally laparoscopic distal gastrectomy. (A) The duodenum is divided from the posterior to the anterior wall at an angle of 90°from the usual line. The stomach is divided from the greater to the lesser curvature. Following the gastric resection, small incisions are created along the edge of the remnant stomach and the duodenum. (B) The posterior walls of the stomach and the duodenum are anastomosed using a linear stapler. (C) Following the formation of a V-shaped anastomosis and an overlap of the enterotomy along the short axis (yellow thick arrows). (D) Closure of the incision using a linear stapler. Addition of the last closure of the line to a V-shaped anastomotic line creates a delta-shape.

## Data Availability

All data supporting the conclusions of this study are included in this published article.

## Abbreviations

CT: Computed tomography
EBD: Endoscopic balloon dilatation

## References

[1] Kanaya S, Kawamura Y, Kawada H, Iwasaki H, Gomi T, Satoh S (2011). The delta-shaped anastomosis in laparoscopic distal gastrectomy: analysis of the initial 100 consecutive procedures of intracorporeal gastroduodenostomy. Gastric Cancer.

[2] Matsuhashi N, Yamaguchi K, Okumura N, Tanahashi T, Matsui S, Imai H (2017). The technical outcomes of delta-shaped anastomosis in laparoscopic distal gastrectomy: a single-center safety and feasibility study. Surg Endosc.

[3] Lee HJ, Park W, Lee H, Lee KH, Park JC, Shin SK (2014). Endoscopy-guided balloon dilation of benign anastomotic strictures after radical gastrectomy for gastric cancer. Gut Liver.

[4] Hanaoka N, Ishihara R, Motoori M, Takeuchi Y, Uedo N, Matsuura N (2018). Endoscopic balloon dilation followed by Intralesional steroid injection for anastomotic strictures after Esophagectomy: a randomized controlled trial. Am J Gastroenterol.

[5] Shoji H, Yamaguchi N, Isomoto H, Minami H, Matsushima K, Akazawa Y (2014). Oral prednisolone and triamcinolone injection for gastric stricture after endoscopic submucosal dissection. Ann Transl Med.

[6] Satomi D, Sakakibara M (2018). Successful treatment by steroid Administration for Four Cases of anastomotic stenosis following Colon surgery. Nippon Daicho Komonbyo Gakkai Zasshi.

[7] Tsuneda A, Nagura S, Maruzen S, Kadoya N. A case of anastomotic stenosis occurred despite the use of 2 different anastomosis techniques in the lower gastrointestinal tract surgery that was effective in corticosteroid use. J Clin Surg. 2019. (In Japanese). 10.11477/mf.1407212486.

[8] Fujihata S, Yamamoto M, Nonoyama K, Watanabe K, Kitagami H (2017). A case of anastomotic stenosis following laparoscopic distal gastrectomy with delta-shaped gastroduodenal anastomosis. J Japan Soc Endosc Surg.

[9] Kochhar R, Makharia GK (2002). Usefulness of intralesional triamcinolone in treatment of benign esophageal strictures. Gastrointest Endosc.

[10] Kim KH, Kim MC, Jung GJ (2012). Risk factors associated with delayed gastric emptying after subtotal gastrectomy with Billroth-I anastomosis using circular stapler for early gastric cancer patients. J Korean Surg Soc.

[11] Hwang SH, Lee JH, Kim BG, Kim SW, Kang JM (2015). The efficacy of steroids for edema and ecchymosis after rhinoplasty: a meta-analysis. Laryngoscope.

[12] Kargi E, Hoşnuter M, Babucçu O, Altunkaya H, Altinyazar C (2003). Effect of steroids on edema, ecchymosis, and intraoperative bleeding in rhinoplasty. Ann Plast Surg.

[13] Yaguchi D, Ichikawa M, Shizu M, Inoue N, Kobayashi D, Imai N (2018). Bronchoscopic local steroid spray to prevent bronchial tuberculosis-induced cicatricial bronchial stenosis: a case series. Medicine.

