# Supplementary figures and images for: Systemic steroid application for treatment of edematous anastomotic stenosis following delta-shaped anastomosis in laparoscopic distal gastrectomy: a case report
Source: BMC Surg. 2020 Jul 22;20:163. doi: 10.1186/s12893-020-00827-3 (PMC7376961; doi:10.1186/s12893-020-00827-3)

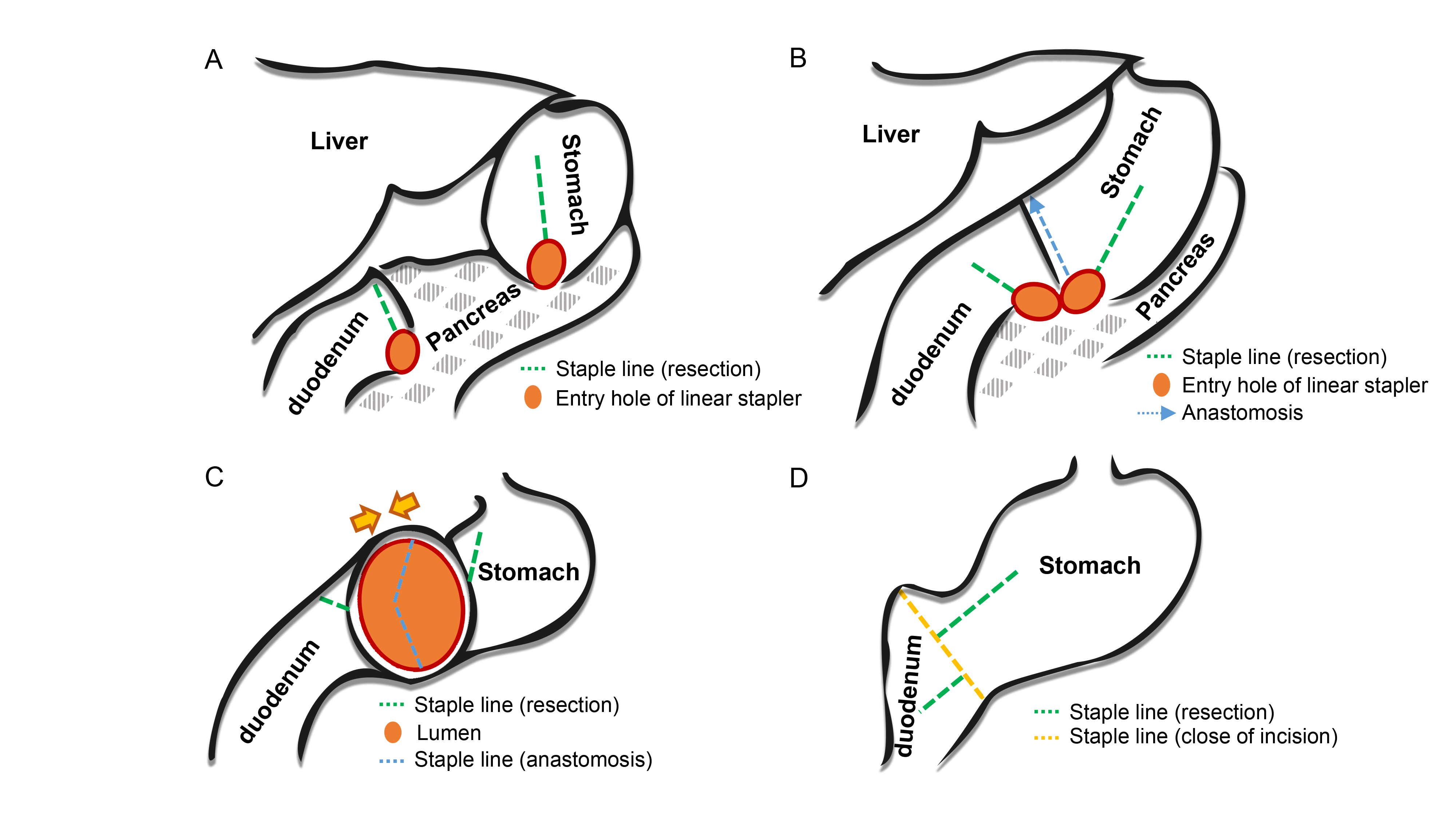

Supplement: Supplementary file 1 — Additional file 1. The illustration of delta-shaped anastomosis in totally laparoscopic distal gastrectomy. (A) The duodenum is divided from the posterior to the anterior wall at an angle of 90°from the usual line. The stomach is divided from the greater to the lesser curvature. Following the gastric resection, small incisions are created along the edge of the remnant stomach and the duodenum. (B) The posterior walls of the stomach and the duodenum are anastomosed using a linear stapler. (C) Following the formation of a V-shaped anastomosis and an overlap of the enterotomy along the short axis (yellow thick arrows). (D) Closure of the incision using a linear stapler. Addition of the last closure of the line to a V-shaped anastomotic line creates a delta-shape. [file 12893_2020_827_MOESM1_ESM.tif]
